# Stool Withholding at School Among Children in the Netherlands

**DOI:** 10.1001/jamanetworkopen.2026.12390

**Published:** 2026-05-13

**Authors:** Sophia P. van Streun, Anne C. ter Schure, Marianne Rook, Bart Sandberg, Joep P. M. Derikx, Marc A. Benninga, Ilan J. N. Koppen, Mariël C. H. Croon, Ramon R. Gorter

**Affiliations:** 1 Department of Pediatric Surgery, Amsterdam University Medical Center location University of Amsterdam, Amsterdam, Noord-Holland, the Netherlands; 2 Department of Pediatric Gastroenterology and Nutrition, Follow Me program, Amsterdam University Medical Center location University of Amsterdam, Amsterdam, Noord-Holland, the Netherlands; 3 Amsterdam Gastroenterology Endocrinology and Metabolism Research Institute, Amsterdam, Noord-Holland, the Netherlands; 4 Amsterdam Reproduction and Development Research Institute, Amsterdam, Noord-Holland, the Netherlands; 5 Dutch Digestive Health Fund (MDL Fonds), Amersfoort, Utrecht, the Netherlands; 6 Verian, Amsterdam, Noord-Holland, the Netherlands; 7 Department of Pediatric Gastroenterology and Nutrition, Amsterdam UMC location University of Amsterdam, Noord-Holland, Amsterdam, the Netherlands

## Abstract

**Importance:**

Understanding toilet avoidance and stool withholding at school is essential, as this can adversely affect children’s health and well-being.

**Objective:**

To assess the prevalence of toilet avoidance and stool withholding at school, and to explore the differences of withholding behavior by gender and geographic location of the school, identify reasons for withholding, and describe physical symptoms and health care utilization.

**Design, Setting and Participants:**

This cross-sectional study used an online questionnaire developed by experts from Amsterdam University Medical Center and the Dutch Digestive Health Fund. Children aged 8 to 16 years from primary schools (aged 8-12 years) and high schools (aged 13-16 years) were recruited by Verian, an external research agency, through a nationwide online panel in the Netherlands.

**Exposure:**

In July 2024, school-aged children completed the questionnaire.

**Main Outcomes and Measures:**

The primary outcomes were the prevalence of stool withholding, reasons for withholding, gastrointestinal symptoms, and health care utilization. Secondary outcomes included differences between gender and geographic location of the school, children’s reasons for avoiding school toilets, and toilets’ perceived cleanliness. To assess potential differences in withholding behavior according to gender and geographic location, a Cochran-Armitage test was performed.

**Results:**

A total of 1000 children, 518 aged 8 to 12 years (264 [51.0%] male) and 482 aged 13 to 16 years (234 [48.5%] male) completed the questionnaire. Stool withholding was reported by 265 primary school children (51.2%) and 344 high school children (71.4%), with no significant differences by gender or geographic location. Hygiene and privacy concerns were the most commonly reported reasons for withholding stool with 610 (84.3%) of children accounting for hygiene and 574 (79.3%) citing privacy concerns, and 410 (41.0%) of children rated school toilet cleanliness as insufficient. Abdominal pain was frequently reported (365 of 724 [50.4%]) and 15 of 336 primary school children (4.5%) experienced fecal incontinence. Overall, 42 primary school children (12.5%) had consulted a physician for symptoms related to withholding.

**Conclusions and Relevance:**

In this cross-sectional study, toilet avoidance and stool withholding at school were common among both primary and high school children, mostly due to hygiene and privacy concerns. Improving school toilet facilities may reduce withholding behavior, gastrointestinal symptoms, and related physician visits, thereby supporting children’s physical, psychological, and educational well-being.

## Key Points

**Question:**

What is the prevalence of and what are the reasons for toilet avoidance and stool withholding at school among children in the Netherlands?

**Findings:**

In this nationwide cross-sectional study of 1000 children aged 8 to 16 years, stool withholding at school was reported by 51.2% of primary school children and 71.4% of high school students, mainly due to privacy, hygiene, and taboo. There were no significant differences by gender or school location.

**Meaning:**

These findings suggest that improving hygiene and privacy of school toilet facilities may reduce stool withholding and related gastrointestinal symptoms, supporting children’s health and well-being.

## Teaser

This cross-sectional study investigates the prevalence of and reasons for toilet avoidance and stool withholding among school-aged children in the Netherlands.

## Introduction

School toilet facilities play a crucial role in children’s daily lives and are highly influential for a child’s well-being. Nevertheless, a growing body of research suggests that school-aged children frequently avoid the toilet at school, particularly for defecation.^1,2,3,4,5^ Reasons for children to develop withholding behavior are frequently related to toilet facility issues, such as cleanliness, privacy, size of the cubicle, and number of toilets available.^1,2,4,6^ Psychological factors, including fear of being bullied, embarrassment, and perceived social stigma, may further discourage children from using the toilet for this purpose.^2,3^ Additionally, the attitudes of parents and teachers toward toilet use, awareness of hygiene, and education about proper toileting may affect children’s perceptions and behaviors regarding toilet use.^5,7,8^ This withholding behavior might result in infrequent and painful defecation, abdominal pain, or even fecal incontinence.^2,9^ Consequently, these symptoms may impair psychosocial functioning, increase school absenteeism and parental stress, and lead to higher health care utilization.^8,10,11^

Despite previously identified associations with withholding behavior and the recognized importance of accessible toilet facilities, school toilets frequently fail to meet children’s expectations.^6^ Previously, our group studied the experiences of parents of children born with anorectal malformations regarding school toilet facilities and identified clean toilets as a protective factor against negative toilet experiences.^12^ Because these were parental perspectives from children born with anorectal malformations, the question arose if this perception differed from the children’s perceptions in the Dutch general population.

Therefore, this study primarily aims to assess the prevalence of toilet avoidance and withholding stool at school among children aged 8 to 16 years. Secondary objectives include exploring reasons for withholding stool, perceptions of cleanliness, associated physical symptoms, and health care utilization related to gastrointestinal symptoms.

## Methods

### Study Design

This cross-sectional study was documented according to the Strengthening the Reporting of Observational Studies in Epidemiology (STROBE) reporting guidelines. An online questionnaire (eMethods in Supplement 1) was used to collect data of children in the Dutch general population from July 8 up to July 16, 2024. A panel of experts from the Amsterdam Pediatric Abdominal Center of the Emma Children’s Hospital together with representatives from the Dutch Digestive Health Fund (MDL Fonds) developed the questionnaire, consisting of 11 questions in 5 domains. Domains of interest were general characteristics, school facilities, toilet behavior, gastrointestinal symptoms, and health care visits. Ethical approval was collected within the Amsterdam University Medical Center.

### Participant Recruitment

An independent external research agency, Verian, reviewed and refined the questionnaire to ensure clarity and suitability for the general population. Where necessary, questions were adjusted to improve comprehensibility. Verian utilized the NIPObase, its online panel comprising over 100 000 individuals from 59 000 households, with regularly updated and detailed sociodemographic data. Participants from the online panel are rewarded with points. These points can be converted into gift cards or air miles. Participants were selected from the NIPObase. Parents in the NIPObase were approached to ask whether children wanted to participate. For the participation of children, an additional consent form was sent by email. Children aged 8 to 16 years living in the Netherlands were eligible for participation. Participants were categorized according to their age into the primary school group (aged 8-12 years) and high school group (aged 13-16 years).

For this study, a stratified sample with quotas was drawn to ensure representativeness across the key demographic variables (place and/or region, age, and gender) relevant to the target age group (primary school, 8-12 years, and high school, 13-16 years). Within each of the 2 age groups, age and gender were interlocked to ensure that both boys and girls were proportionally represented across each age group. Responses were excluded when questionnaires were not fully answered, or quality of the answers was not sufficient (eg, straight lining, extremely fast response time, or double responses).

To assess the clarity and completeness of the questionnaire, it was initially distributed to 100 participants, after which a quality check was performed by Verian. The full sample was invited, and reminders were sent out on July 12, 2024, to boost response rates. To reach the final target of 1000 completed questionnaires, an additional sample was invited on July 15. Answers were completely anonymous and could not be traced back. To improve the response rate, the questionnaire was designed to be simple and concise, with closed answers and with an average estimated completion time of 5 to 7 minutes.

### Outcomes and Measurements

Questions were multiple choice or were based on a scale, in which 1 was the possible lowest, and 10 the possible highest. To accommodate the sensitive nature of some of the questions, which pertained to toilet use at school, parents were given the choice of either completing the questionnaire together with their child or granting permission for the child to complete it independently. The baseline characteristics collected were age in years, gender, and geographic location distinguished for province or big cities and number of addresses nearby.

The primary outcome of this study was the percentage of children who used the school toilet facilities for urination, defecation, or both. Secondary outcomes included differences between gender and geographic location of the school, children’s reasons for avoiding school toilets, and perceived cleanliness of school toilets, rated 1 to 10. Following the Dutch grading system, scores lower than 5.5 were considered insufficient. Furthermore, whenever children responded that they withhold defection at school always, mostly, sometimes, or almost never, a follow-up question was proposed. The follow-up questions pertained to the presence of gastrointestinal symptoms associated with withholding and the frequency of physician visits related to symptoms of withholding stool.

### Statistical Analysis

Statistical analysis was performed using SPSS Statistics for Windows, version 28 (IBM Corp). Descriptive statistics are presented as frequencies with percentages. To assess potential differences in withholding behavior according to gender and geographic location, a Cochran-Armitage test was performed. Statistical differences were considered significant if *P* < .05 (2-tailed).

## Results

A total of 2750 participants from the NIPObase panel were invited for participation, of whom 1087 respondents filled in the questionnaire (response rate 39.5%). After data checks, 2 responses were omitted due to quality concerns because of extremely fast responses. Additionally, there were 85 (7.8%) incomplete responses, resulting in 1000 valid complete responses (36.4%) for the final analysis. The response was split between the 2 age groups: 518 respondents in the primary school group (8-12 years) and 482 respondents in the high school group (13-16 years). In the age group 8 to 12 years, 458 (88.4%) completed the questionnaire together with their parent, and 313 participants (64.9%) in the group aged 13 to 16 years. Gender was distributed evenly in both groups, with 264 (51.0%) male primary school students and 234 (48.5%) male high school students (Table 1).

**Table 1.** Baseline Characteristics

Characteristics	Children, No. (%)	
	Aged 8-12 y (n = 518)	Aged 13-16 y (n = 482)
Gender		
Male	264 (51.0)	234 (48.5)
Female	253 (48.8)	246 (51.0)
Missing	1 (0.2)	2 (0.4)
Questionnaire completed with a parent	458 (88.4)	313 (64.9)
Geographic location		
Very strong urbanization	114 (22.0)	72 (14.9)
Strong urbanization	176 (34.0)	178 (36.9)
Moderate urbanization	78 (15.1)	87 (18.1)
Little urbanization	114 (22.0)	106 (22.0)
No urbanization	36 (6.9)	39 (8.1)
Prevalence of toilet avoidance and stool withholding at school		
Urinate at school		
Yes, always	304 (58.7)	201 (41.7)
Yes, mostly	185 (35.7)	214 (44.4)
No	26 (5.0)	64 (13.3)
I do not know	3 (0.6)	3 (0.6)
Defecate at school		
Yes, always	154 (29.7)	50 (10.4)
Yes, mostly	97 (18.7)	49 (10.2)
Sometimes	82 (15.8)	76 (15.8)
Almost never	90 (17.4)	116 (24.1)
No, never	91 (17.6)	188 (39.0)
I do not know	4 (0.8)	3 (0.6)
Stool withholding at school		
Yes, always	51 (9.8)	102 (21.2)
Yes, mostly	64 (12.4)	105 (21.8)
Sometimes	150 (29.0)	137 (28.4)
Almost never	71 (13.7)	44 (9.1)
No, never	22 (4.2)	36 (7.5)
I do not know	2 (0.4)	5 (1.0)
Missing	158 (30.5)	53 (11.0)

In the primary school group, 489 children (94.4%) indicated that they always or mostly urinated at school. For the high school group, this percentage was 86.1% (415 children) (Table 1). Twenty-six children (5.0%) of the primary school group and 64 children (13.3%) in the high school group never urinated during school hours. Regarding defecation, 181 children (34.9%) of the primary school group reported to never or almost never use school toilet facilities for defecation. This percentage was higher in the high school group (304 children [63.1%]). Furthermore, 265 children (51.2%) in the primary school group and 344 children (71.4%) in the high school group reported that they always, mostly, or sometimes withhold defecation during school hours. These patterns of withholding stool were evenly distributed between groups for gender; 170 male children (50.6%) in the primary school group and 189 male children (48.7%) in the high school group reported stool withholding. No significant differences were found between gender (male or female) and geographic location of the school (very strong urbanization*, *strong urbanization, moderate urbanization, little urbanization, and no urbanization) (Table 2).

**Table 2.** Stool Withholding at School Stratified for Gender and Geographic Location of the School, With Cohran-Armitage Test Per Age Category

Characteristic	Aged 8-12 y (n = 518)		Aged 13-16 y (n = 482)	
	Children, No. (%)	*P* value	Children, No. (%)	*P* value
				
Gender differences, No. female/No. male (female, %)	253/264 (48.8)	.65	246/234 (51.0)	.80
Geographic differences				
Very strong urbanization	70 (20.8)	.63	65 (16.8)	.46
Strong urbanization	114 (33.9)		135 (34.8)	
Moderate urbanization	60 (17.9)		71 (18.3)	
Little urbanization	69 (20.5)		83 (21.4)	
No urbanization	23 (6.8)		34 (8.8)	

The Figure shows the most frequently reported reasons for withholding stool during school hours. These were related to hygiene (610 children [84.3%]) and privacy concerns (574 children [79.3%]) for both age groups. With increasing age, a higher proportion of children reported emotional factors, such as perceptions of taboo (265 children [68.3%]), feelings of shame (255 children [65.7%]), and concerns about peers (216 children [55.7%]) as reasons for withholding stool during school hours. Overall, the rate for cleanliness of the school toilet facilities had a median (IQR) of 6.0 (4.0-7.0), and 41.0% (410 children) rated cleanliness to be insufficient (<5.5, on a scale of 1 to 10) (eFigure in Supplement 1).

**Figure.**
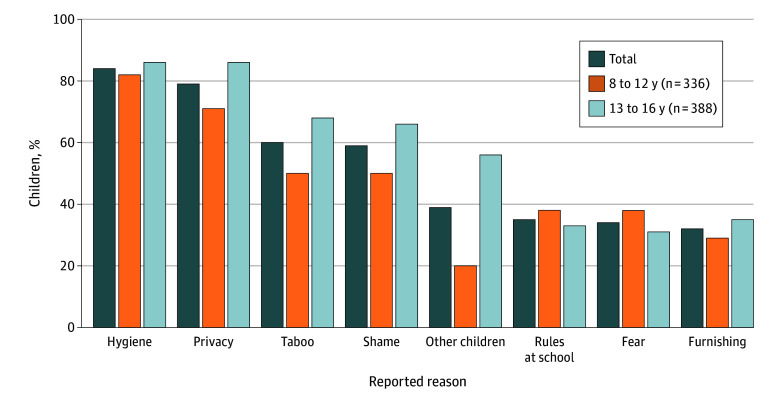
Bar Graph of Reasons for Stool Withholding

Table 3 shows the presence of gastrointestinal symptoms and health care visits among children who reported withholding stool at school. A total of 366 children in primary school and 388 children in high school reported stool withholding. In the primary school group, 175 children (52.1%) reported experiencing abdominal pain related to withholding their stool compared with 190 children (49.0%) in the high school group. The proportion of children reporting no symptoms was 107 (31.8%) in the primary school group and 154 (39.7%) in the high school group. Fecal incontinence was reported by 4.5% of children (15 children) in the primary school group; for the high school group, this was 1.3% (5 children). Overall, 42 children (19.6%) in the primary school group and 29 (7.5%) children in the high school group reported having at least once visited a physician related to these symptoms (Table 3).

**Table 3.** Consequences Associated with Stool Withholding, Stratified Per Age Category^a^

Consequence	Children, No. (%)	
	Aged 8-12 y (n = 336)	Aged 13-16 y (n = 388)
Physical symptoms associated with stool withholding		
Abdominal pain	175 (52.1)	190 (49.0)
Flatus and distended abdomen	67 (19.9)	81 (20.9)
Nausea	16 (4.8)	32 (8.2)
Constipation	64 (19.0)	62 (16.0)
Involuntary loss of stool	15 (4.5)	5 (1.3)
Involuntary loss of urine	12 (3.6)	6 (1.5)
Urinary tract infection	3 (0.9)	5 (1.3)
No symptoms	107 (31.8)	154 (39.7)
I do not know	16 (4.8)	14 (3.6)
Physician visits associated with symptoms of stool withholding		
Yes, multiple times	23 (6.8)	17 (4.4)
Yes, once	19 (5.6)	12 (3.1)
Not yet, but I am thinking about it	7 (2.1)	3 (0.8)
Not yet	45 (13.4))	51 (13.1)
No, not necessary	119 (35.4)	137 (35.3)
Missing	123 (36.6)	168 (43.3)

^a^ This follow-up question was presented to children who reported stool withholding as always, mostly, sometimes, or almost never.

## Discussion

This cross-sectional study presents data on the prevalence of toilet avoidance, as well as stool withholding and its underlying motivations, among children aged 8 to 16 years in the Netherlands. We found that up to 34.9% of children in the primary school group (8-12 years) and 63.1% of the children in the high school group (13-16 years) never defecate during school hours. Children who withhold defecation during school times frequently report abdominal pain, nausea, and symptoms of constipation. Related to these symptoms, 42 children in primary school (12.5%) and 29 in high school (7.5%) visited a physician. Moreover, in the primary school group, almost 5.0% of the children reported fecal incontinence. Most frequently reported reasons for children that withhold their defecation were poor hygiene (84.3%) and lack of privacy (79.3%) in school toilet facilities. Although median (IQR) toilet cleanliness was rated a 6.0 (4.0-7.0), 41.0% of respondents rated cleanliness as insufficient. In older children, psychosocial factors such as shame (65.7%) and societal taboos (68.3%) became increasingly more important.

Refraining from defecating at school is consistent in previous studies conducted in comparable populations of children aged 6 to 18 years.^3,5^ A quantitative questionnaire study including 385 Norwegian children, aged 6 to 16 years, found that up to 63.0% never defecated at school.^3,13^ In line with our results, reasons for avoidance included smell, cleanliness, and concerns about safety associated with a lack of privacy. Another study surveyed 102 children, aged 6 to 12 years, and identified cleanliness and limited time to use the bathroom as main factors contributing to withholding behavior.^5^

In addition, inadequate toilet numbers and doors that did not close properly are environmental factors that have been associated with withholding behavior.^3^ It is well known that environmental stimuli influence children’s perceptions and their experience of privacy, which has been associated with stool withholding and functional constipation in approximately 30.0% of primary school children.^2,3^ Furthermore, privacy concerns can increase discomfort and anxiety, deterring toilet use during school hours.^6^ Beyond physical factors, toilet avoidance is shaped by cultural norms and social context, including shared expectations around hygiene, privacy, and peer behavior. Cultural and socioeconomic differences, even within high-income countries, are also associated with an elevated risk of functional constipation.^3,14,15,16^

In addition, gastrointestinal symptoms prompted physician visits in 42 children aged 8 to 12 years (12.5%) and 29 children aged 13 to 16 years (7.5%). However, these symptoms cannot be solely attributed to toilet avoidance, but can be a cause or consequence of preexisting constipation; psychological factors, diet, sleep, and functional gastrointestinal disorders may also contribute to these symptoms.^17^ To mitigate confounding, children reported only symptoms they linked to stool withholding.

Hence, we cannot establish a definitive causal relationship between negative toilet experiences and gastrointestinal symptoms. Furthermore, the diagnosis of constipation was not confirmed (for example by using the Rome IV criteria^18^), but was based on the participants’ interpretation. However, it is important to gain a clear understanding of the risk of constipation, given its potential for long-term consequence. Childhood constipation can persist into adulthood, with approximately one-third of affected children continuing to experience similar issues later in life.^17,19^ A systematic review on the quality of life of children with functional constipation found a marked reduction in quality of life, with adverse associated effects persisting into adolescence in approximately 25.0% of cases. These long-term consequences were partly attributed to the psychological comorbidities that frequently accompany functional constipation, suggesting that the burden may extend beyond childhood.^20^

It is important to note that 4.5% of children in the primary school group are affected by fecal incontinence. A Danish study surveyed 19 577 healthy children regarding toilet avoidance and its consequences. Their findings highlighted an association between fecal incontinence, toilet avoidance, and dissatisfaction with school toilet facilities.^21^ Fecal incontinence is frequently associated with a reduced quality of life related to psychological problems.^21,22,23^ In addition, fecal incontinence has been associated with increased school absenteeism, which may contribute to educational difficulties.^8^ Although the prevalence of fecal incontinence may appear low, a rate of 4.5% suggests that nearly every primary school classroom contains at least 1 affected child.

Improving hygiene in school toilet facilities could be a straightforward step to increase their use, as unclean restrooms are linked to stool withholding.^9,10,11,24^ Responsibility often lies with individual schools, and Dutch regulations are limited and unenforced.^25^ International initiatives, such as student-led refurbishments in Australia and structured hygiene programs in Laos, have increased toilet use (37.0%), indicating that multifaceted, enforceable interventions may be required.^26,27^ Although school toilet facilities in Laos differ substantially from those in Western countries, a similar improvement strategy may be necessary to promote toilet use. In the Netherlands, adaptations to school toilet facilities remain limited, as existing guidelines are not legally binding and are not subject to routine inspection or enforcement. The lack of comprehensive, standardized regulations may result in disparities in school toilet facility quality, possibly due to limited school budgets. Future research should examine barriers to implementation and identify best practices from schools with well-maintained facilities. While policy-level improvements are important, parental guidance and established toileting habits at home may also shape children’s toilet behavior at school.

The results of this study should be interpreted in light of its strengths and limitations. Strengths include its representative sample, which involved schools from across the country in both urban and village areas and its large sample size (including 1000 respondents). Addressing this emerging topic is highly relevant for public health, particularly in the context of increasing use of laxatives among children in Europe.^24^ To our knowledge, this study provides the first nationwide estimates of school toilet avoidance and stool withholding and, therefore, establishes an important foundation for future research and, eventually, targeted interventions. Furthermore, to our knowledge, this is the first study to account for age, gender, and geographic differences in the analysis of reasons for avoiding school toilets, associated gastrointestinal symptoms, and physician visits related to these symptoms.

Further studies should explore strategies to reduce withholding behavior during school hours. Stakeholders should be informed and involved in the design and implementation of such strategies. These findings provide valuable information to address and discuss these problems, with the overall aim of improving school toilet facilities and thereby stool withholding during school hours. This in turn might also be associated with a reduction in the reported gastrointestinal symptoms and physician visits by reducing stool withholding.

### Limitations

This study has several limitations. First, only children aged 8 to 16 years were included, limiting generalizability to younger children (aged 4-7 years), for whom appropriate school toilet facilities may be even more important. Second, data were collected via a self-reported questionnaire that has not been previously validated. Although developed by experts from a tertiary medical center in collaboration with the MDL Fonds, the use of nonvalidated items may have introduced variation in the interpretation of terms such as almost, and participants’ individual definitions were not captured.

Third, the study focused exclusively on school-time toilet behavior and related symptoms, providing limited insight into overall toilet behavior patterns (eg, toilet habits at home). Additionally, reliance on self-reported data may have introduced recall bias, potentially affecting the accuracy of reported behaviors and symptoms, despite administration during the school term. Social desirability bias may have led to underreporting of toilet avoidance, stool withholding, or fecal incontinence. Response bias could also arise, particularly among younger children completing the questionnaire with parental assistance, potentially affecting subjective responses such as perceived toilet cleanliness. Additionally, the lack of third-party assessments introduces measurement bias, as all data reflect children’s and parents’ perceptions rather than objective evaluations. The potential magnitude of these biases is likely moderate, particularly for subjective measures. Causality between gastrointestinal symptoms and stool withholding cannot be established, as such symptoms may occur independently of toilet-related experiences, limiting the ability to attribute these symptoms solely to toilet-related factors. Fourth, participants were recruited via an external agency (Verian) from its database. While participation was incentivized through points unrelated to responses, the external agency provided oversight and ensured quality control, monitoring, and removal of duplicate or rapid responses, reflected in the high completion rate of the questionnaire.

## Conclusions

In this cross-sectional study, children aged 8 to 16 years were found to frequently withhold defecation during school hours, largely driven by concerns regarding hygiene and privacy. The cleanliness of school toilet facilities was often perceived as insufficient. Newly targeted interventions to improve school toilet environments should be developed to support children’s health and well-being.

## Notes

Supplement 1. eMethods. Questionnaire eFigure. Figuratively displayed grades of school toilets

Supplement 2. Data Sharing Statement
