# Supplementary material for: Stool Withholding at School Among Children in the Netherlands
Source: JAMA Netw Open. 2026 May 13;9(5):e2612390. doi: 10.1001/jamanetworkopen.2026.12390 (PMC13173384; doi:10.1001/jamanetworkopen.2026.12390)
Supplement: Supplement 1. — eMethods. Questionnaire eFigure. Figuratively displayed grades of school toilets [file jamanetwopen-e2612390-s001.pdf]

Supplemental Online Content

van Streun SP, ter Schure AC, Rook M, et al. Highlighting the problem of stool withholding at school among children in the Netherlands. *JAMA Netw Open.* 2026;9(5):e2612390. doi:10.1001/jamanetworkopen.2026.12390

eMethods. Questionnaire
eFigure. Figuratively displayed grades of school toilets

This supplemental material has been provided by the authors to give readers additional information about their work.

© 2026 van Streun SP et al. *JAMA Network Open*.

eMethods. Questionnaire

Guardian status

1. Are you the legal guardian of one or more children or adolescents (aged 8–16 years) living in your household?
Parents are automatically the legal guardians of their children. In some cases, there may be exceptions, for example, when a court has decided otherwise.

Completion of the questionnaire
2. Are you completing this questionnaire together with your child, or do you give permission for your child to complete it independently?

Demographics
3. How old are you?
4. What is your gender?

Toilet use at school
5. When you are at school, do you go to the toilet when you need to urinate?
6. When you are at school, do you go to the toilet when you need to defecate?
7. Do you ever hold back your stool when you are at school?

Reasons for avoiding the toilet at school
8. The toilets smell bad.
9. The toilets are dirty.
10. You are only allowed to go during breaks, when it is too crowded.
11. You can easily hear other people in the toilet.
12. The toilets and sinks are old.
13. Other students vape in the toilets.
14. Other students smoke in the toilets.
15. The toilets are used for other purposes.
16. I was afraid that the toilet door would not open.
17. I was afraid that other children could open the door from the outside.
18. I was not allowed to go by the teacher.
19. Toilet paper, soap, or other necessities were not available.
20. I was bullied in or near the toilets.
21. There were no separate toilets for boys and girls.
22. I felt ashamed because of smells, sounds, or other reasons.
23. I simply do not use the toilet at school.

Physical complaints associated with holding back stool
24. Have you ever experienced any of the following complaints when you held back your stool? (You may select multiple options.)

- Abdominal pain
- Flatulence or bloating
- Nausea
- Constipation or hard stools
- Concentration problems
- Involuntary loss of stool
- Involuntary loss of urine
- Urinary tract infection
- Other, namely: ___________

© 2026 van Streun SP et al. *JAMA Network Open*.

- No, I have never experienced any complaints
- I don’t know

Frequency of complaints
25. How often have you experienced these complaints when you held back your stool?

Medical consultation
26. Have you ever visited a doctor because of these complaints that occurred after holding back your stool?

Cleanliness rating
27. How clean do you consider the toilets at your school? *(Please provide a rating, scale from 1-10.)*

Open-ended question
28. Do you have any suggestions for improving toilet use at schools?
For example: playing calm music, self-cleaning toilets, soundproofing materials, or automatic air fresheners.

© 2026 van Streun SP et al. *JAMA Network Open*.

eFigure. Figuratively displayed grades of school toilets

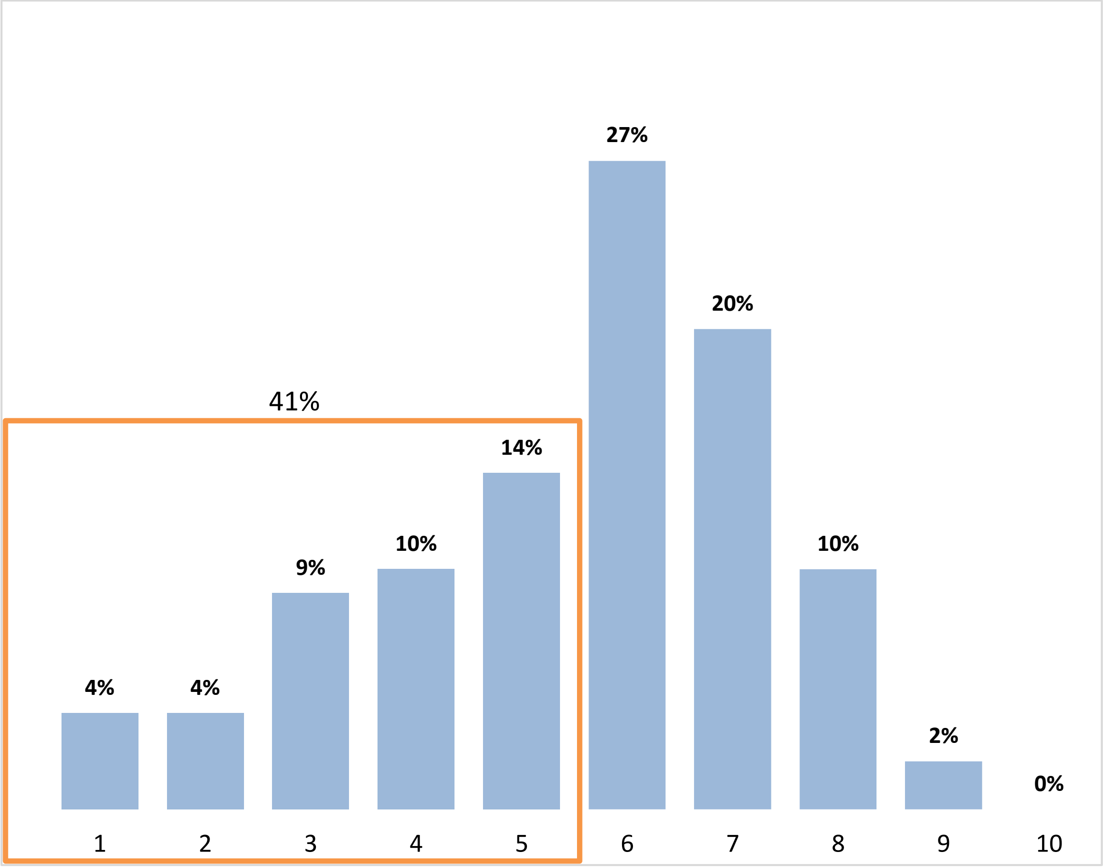

© 2026 van Streun SP et al. *JAMA Network Open*.
