# Supplementary material for: Stool Withholding at School Among Children in the Netherlands
Source: JAMA Netw Open. 2026 May 13;9(5):e2612390. doi: 10.1001/jamanetworkopen.2026.12390 (PMC13173384; doi:10.1001/jamanetworkopen.2026.12390)
Supplement: Supplement 2. — Data Sharing Statement [file jamanetwopen-e2612390-s002.pdf]

Data Sharing Statement

van Streun. Highlighting the Problem of Stool Withholding at School Among Children in the Netherlands. *JAMA Netw Open*. Published May 13, 2026.
doi:10.1001/jamanetworkopen.2026.12390

Data
Data available: No
